# Risk Factors Related to Peripherally Inserted Central Venous Catheter Nonselective Removal in Neonates

**DOI:** 10.1155/2018/3769376

**Published:** 2018-05-30

**Authors:** Xiaohe Yu, Shaojie Yue, Mingjie Wang, Chuanding Cao, Zhengchang Liao, Ying Ding, Jia Huang, Wen Li

**Affiliations:** Department of Paediatrics, Xiangya Hospital of Central South University, Changsha, Hunan 410008, China

## Abstract

We aimed to investigate the incidence and risk factors associated with nonselective removal of peripherally inserted central venous catheter (PICC) in neonates. In this prospective cohort study, neonates who underwent PICC placement at neonatal intensive care units (NICUs) in China from October 2012 to November 2015 were included. The patient demographics, catheter characteristics, catheter duration, PICC insertion site, indication for PICC insertion, infuscate composition, PICC tip location, and catheter complications were recorded in a computerized database. Risk factors for nonselective removal were analyzed. A total of 497 PICCs were placed in 496 neonates. Nonselective removal occurred in 9.3% of PICCs during 10,540 catheter-days (4.6 nonselective removals per 1,000 catheter-days). These included occlusion (3%), infection (1.4%), leakage (2.0%), phlebitis (0.6%), displacement (1%), pleural effusion(0.6%), and breaks (0.6%). Noncentral tip position was independently associated with an increased risk of nonselective removal (odds ratio 2.621; 95% confidence interval, 1.258-5.461) after adjusting for gestational age, sex, birth weight, and PICC dwell time. No significant differences in the rate of complications occurred between silastic and polyurethane PICC or different insertion sites. Noncentral PICC tip position was the only independent risk factor for nonselective removal of PICC.

## 1. Introduction

Peripherally inserted central catheters (PICCs) are routinely used in term and preterm infants to provide intravenous access for prolonged therapy and parenteral nutrition [1, 2]. They are known to reduce the complications associated with the conventionally used central catheters. PICCs can be conveniently inserted at the bedside without the need for surgical intervention. They are essential in delivering life-saving treatment to neonates. In particular, PICCs represent a large proportion of central lines inserted in the neonatal intensive care unit (NICU).

PICCs are associated with a reduced incidence of complications such as thrombosis, catheter occlusion, and leakage compared to short peripheral catheters [3]. Despite these advantages, PICCs are associated with various complications such as occlusion, infection, thrombosis, breakage, migration, and displacement [4, 5], which lead to nonselective removal of the catheter. Despite the studies illustrating the advantages associated with PICC use, short peripheral catheters are vastly used in neonatal intensive care units (NICUs) for long-term intravenous therapies, total parenteral nutrition, and drug injection in Iran, causing an increased incidence of catheterization and complications, which can be prevented by using PICCs [6]. The daily risk of infection was reported to be higher in PICCs in place for >2 weeks than PICCs that were used for <2 weeks [7]. As these complications are associated with morbidity among neonates, therefore, clinical data on nonselective removal may help in quality improvement efforts [8, 9].

Previous studies have identified risk factors for complications of PICCs in neonates. These risk factors included young age, severity of illness, catheter dwell time, catheter tip position, and catheter insertion site [8, 10–14], but reports in Chinese neonates are rare. Identifying modifiable risk factors of complications is especially important as clinicians work to prevent catheter complications.

Therefore, in the present study, we aimed to determine the association between patient and catheter characteristics and the risk of nonselective removal in newborns.

## 2. Methods

Between October 2012 and November 2015, 496 consecutive infants who had been admitted to the 60-bed neonatal Intensive Care Unit (ICU) of Xiangya Central South University (China) and underwent insertion of a PICC were included in the study. Styptic was indicated for patients who required parenteral nutrition support for more than 1 week. Informed consent was obtained from the parents of all the infants prior to the start of the study.

All PICCs were placed by a specialized team of nurses under the supervision of pediatric interventional radiologists. The upper limb approach was preferred in most cases because there are more veins available and the distance from the vena cava is shorter. After PICC placement, all catheter tip positions were determined by brief fluoroscopy. Catheter tips were defined as central at the time of placement if they were in the superior vena cava (SVC), right atrium (RA), or high inferior vena cava (IVC), at or above the level of the diaphragm, and as noncentral if located elsewhere. The PICCs were removed in the case of adverse events.

Data regarding the patient's age, sex, indications for catheter insertion, catheter characteristics, catheter duration, infusate composition, and catheter complications were entered prospectively into a computerized database. PICC insertion sites were categorized into different blood vessels of upper extremity, lower extremity, and head and neck.

Silastic catheters (1.9 Fr, BD USA) were exclusively used during the first two years of this study. A total of 345 silastic PICC lines were placed in the NICU, but we stopped using these catheters because BD Biosciences stopped the production of the silastic catheters. From October 2014 onwards, 152 polyurethane PICC lines of the same size (1.9 Fr, Medcomp, USA) were used. All PICCs were flushed using heparin sodium.

Data regarding all nonselective removals were collected from all the neonates. The complications included leakage at the PICC insertion site, phlebitis (erythema, swelling, pain, or palpable cord), infection (positive blood and catheter tip cultures), catheter occlusion (inability to infuse or withdraw), and mechanical malfunction (catheter damage). Catheters were removed after completion of therapy or in case of complications. The primary outcome was defined as the presence of complications leading to PICC nonelective removal. The time at risk for complications was the PICC dwell time, calculated as the number of days between insertion and removal of PICC.

### 2.1. Statistical Analysis

All analyses were performed using the SAS statistical software, version 7 (SAS Institute Inc., Cary, NC). Nonnormally distributed continuous variables were expressed as median (IQR). Categorical variables were expressed as frequencies. For simple comparisons between central and noncentral PICCs, the chi-square test and Fisher's exact test were used to analyze categorical data. The nonparametric test was used for continuous variables. Odds ratio (OR) was calculated using multiple logistic regression with adjustments for gestational age, sex, birth weight, and PICC dwell time. Statistical significance was defined as P<0.05.

## 3. Results

Between October 2012 and November 2015, data from a total of 497 PICCs were analyzed from 496 patients.  Table 1 presents the patient characteristics. The mean gestational age (GA) was 31.0±2.5 weeks; the mean birth weight was 1537±523 g. There were 284 (57.3%) males and 212 (42.7%) females. Most of the newborns were premature (467, 94.2%). The GA of 31 infants was ≤28 weeks; 28-32 weeks for 312 patients; 32-37 weeks for 124 patients; and >37 weeks for 29 patients. Extremely low birth weight (ELBW) was defined as a birth weight of <1000 g and very low birth weight (VLBW) was defined as a birth weight of <1500 g. Among the neonates in our study, 48 were ELBW and 236 were weighed 1000-1499 g. 152 weighed 1500-2000 g and 57 weighed >2000 g. 2 have no birth weight record.

The catheters were inserted at a median (interquartile range) age of 8 (1-60) days. Parenteral nutrition was the most common indication for PICC, with the majority of infants receiving >12.5% dextrose-based infusion (450 PICCs); 24 infants received <12.5% dextrose-based solutions, and 23 PICCs were placed exclusively for intravenous antibiotic therapy. The majority of PICCs (410, 82.5%) were inserted in the upper extremities (Table 2). The mean duration of PICC was 18 days (range: 0-74 days).

Of the 497 PICCs, 413 (83.1%) were centrally positioned (central group), 83 (16.8%) were noncentrally positioned (noncentral group), and one had no record regarding its position. There were no differences between the two groups regarding GA, birth weight, patient classification, side of PICC insertion, PICC insertion site, and PICC material (all P>0.05). There are more boys in the noncentral PICC group (67.5% vs. 55.2%, P=0.039). The rates of nonselective removal (18.1% vs. 7.5%, P=0.002) were higher in the noncentral group compared with the central group** (Table 1).**

Complications occurred in 9.3% of PICCs over a total of 10,540 catheter-days (4.6 complications per 1,000 catheter-days). These included occlusion (3%), infection (1.4%), leakage (2.0%), phlebitis (0.6%), displacement (1%), pleural effusion(0.6%), and breaks (0.6%). Most of the PICCs (451, 90.7%) were removed electively on completion of therapy, while 46 PICCs (9.2%) were removed due to one of the abovementioned complications. Of these, 39 PICCs were removed secondary to noninfectious complications and 7 were removed for infection-related complications (Table 3).

There were no statistically significant differences between the types of infusates administered to the central and noncentral groups. TPN was the most commonly used infusate in both groups. No statistically significant differences were observed in the GA at insertion (weeks), birth weight, and the mean PICC duration between the central and noncentral groups. There were 31 complications involving 413 centrally positioned catheters (7.5%) and 15 complications among the 83 noncentral catheters (18.1%;* P*=0.002).

The majority of PICCs (69.4%) were silastic, while 30.6% of the PICCs were polyurethane. During the study period, there were three cases (0.6%) of pleural effusion. All three patients had polyurethane PICCs for parenteral nutrition.

The unadjusted (univariate analyses, Table 4) and adjusted (multivariate analysis, adjusted for catheter dwell time, age, insertion site, indication for PICC insertion, and noncentral tip position; Table 5) risk factors for PICC complications showed an increased risk of complications for noncentral PICCs compared to central PICCS (OR: 2.621; 95% CI: 1.258-5.461;* P*=0.01; Table 5).

## 4. Discussion

In the present study, we aimed to characterize and identify the risk factors for complications necessitating removal of PICCs in neonates. Our study findings indicated that noncentrally located PICCs were an independent risk factor for nonselective removal. Despite reductions in the incidence of nonselective removal, further efforts are needed to prevent PICC-associated complications in neonates.

In previous studies, premature birth, severity of infant condition, PICC duration [7, 15] tip position [10, 16], and site of PICC insertion [11–13] have been suggested to be risk factors of nonselective removal. An increased rate of complications has been documented with noncentral PICC tips. Moreover, factors such as small vessel size, decreased blood flow rate, turbulent flow, and endothelial injury are considered to contribute to these complications. In our study, although noncentrally located PICCs represented a small percentage (16.7%) of inserted PICCs. They were more likely to be removed secondary to a complication compared with PICCs with a central tip position.

Previous studies have reported nonselective removal rates ranging 2.9-20.8% [17–20]. The rates of complications in our study are similar to those reported previously. Despite the lower rates of complications associated with centrally located PICCs, often it is not possible to achieve centrally located PICCs due to factors such as venospasm, venous tortuosity, and venous valves [19]. There is no clear evidence in the literature indicating the association of catheter tip position and complication rates in pediatric PICCs. Some pediatric studies have found that PICCs placed in noncentral veins provided safe and reliable intravenous access [17], whereas others have suggested that PICCs terminating in noncentral venous positions have higher risks of complication [10, 16, 19]. Nevertheless, these studies cannot be easily compared due to inconsistent definitions of central veins. Some authors have classified the subclavian vein as a central position for the catheter tip. Most clinicians consider a PICC to terminate in a central vein if the tip is located in the IVC, SVC, or right atrial junction (RAJ). After adjusting for other important predictors of PICC complications such as age, catheter dwell time, PICC insertion site, and indications for PICC insertion, our findings are in line with previous findings that a noncentral catheter tip position is associated with increased rates of nonselective removal.

Increased complication rates in noncentral PICCs, especially mechanical complications, may be the result of a combination of factors such as vessel size, turbulence, blood flow rate, and endothelial injury. A cadaveric study of ELBW infants revealed that the outer diameter of the subclavian veins was significantly smaller than the BC (mean diameters of 2.6 and 2.5 mm vs. 3.3 and 4.0 mm for the right and left, respectively) [21]. The outer diameter of vessels with the catheter tip may be inversely related to the rate of mechanical and infiltrative complications. In another study, the researchers discouraged the insertion of PICC tips in subclavian veins in neonates because catheters located in these veins had a higher rate of infiltration and mechanical complications and shorter time to complications [8]. The results of the present study suggested that noncentral catheters were an independent risk factor for noninfectious complications. Therefore, noncentrally located PICCs should be used with caution due to their increased risk of complication.

Ong et al. carried out a prospective randomized study to compare the complications between polyurethane and silicone PICCs [22]. They randomly assigned 326 patients to a proximal valve polyurethane PICC or a distal valve silicone PICC. Polyurethane PICCs were found to be more durable than silicone PICCs with a significantly lower incidence of complications (26.8% vs. 47.9%; P<0.001), particularly phlebitis and catheter-related infections. No hydrothorax complications occurred in both groups. Hydrothorax complications have been reported to occur regardless of the size or material of the PICC [23]. Nevertheless, Pezzati et al. carried out a retrospective study involving 280 PICCs in 258 preterm neonates and found that no pleural effusion or cardiac tamponades occurred in the silastic PICC group (232/280; 82.9%), whereas there was one case of pleural effusion and five of cardiac tamponades in the polyurethane PICC group (48/280; 17.1%). Based on these results, Pezzati et al. suggested that silastic catheters are safer and should be preferred over polyurethane ones [24]. We found no significant differences in the rate of total complications and catheter-related infections between the two types of PICC catheters, but there was a significantly lower incidence of complications of occlusion, phlebitis, displacement, and breakage and a higher incidence of pleural effusion in polyurethane PICCs. Among the infants who received total parenteral nutrition via PICC, three infants suffered from PICC-induced hydrothorax. Pleural fluid accumulation can occur due to SVC obstruction with obstruction of lymphatic drainage and erosion or perforation of the catheter through the vein into the pleural space. In our experience, all the three neonates initially had polyurethane PICCs in their SVC, but two of the PICCs came out due to migration. We suspect that as polyurethane PICCs are stiffer and less flexible than silastic catheters, they can more easily damage the vascular wall when placed in the SVC due to the curve of the aortic arch. In addition, similar to the previous study, we also suggest choosing silastic catheters over polyurethane ones, especially in the case of ELBW babies. If the use of a polyurethane catheter is unavoidable, we would recommend using the saphenous approach in order to avoid the aortic curve. A large-scale prospective randomized multicenter study is required to evaluate the incidence of pleural effusion and cardiac tamponades in these two types of PICCs.

This study has several limitations. First, this was an observational study and is therefore vulnerable to bias. The line tip position was also not regularly monitored, nor was thrombosis identified via ultrasound. Secondly, some catheter infections may have been treated with antibiotics while the PICC remained in place, and these complications may not have been captured. Finally, despite this being a large cohort, our findings may not be generalizable as this was a single-center study. Larger prospective studies across multiple centers are needed to clarify the relationship between these possible risk factors and PICC complications.

## 5. Conclusion

Our prospective cohort study identified that noncentral catheter tip position was the only independent risk factor for nonselective removal of PICC. Therefore, Clinicians should ensure that catheter tips reside in the RA, IVC, or SVC at or above the level of the diaphragm in neonates.

## Figures and Tables

**Table 1:** Comparison of infant characteristics and outcomes between central and noncentral tip position.

Characteristics	Central (n=413)	Non-central (n=83)	P value
Gestational age at insertion (weeks)	31.0 (24.5,40.0)	31 (26.2,39.5)	0.841
Birth weight (g)	1445 (700,5170)	1450 (680,3980)	0.716
Sex, n (%)			0.039
Boy	228 (55.2%)	56 (67.5%)	
Girl	185 (44.8%)	27 (32.5%)	
Patient classification, n (%)			0.441
Medical	396 (95.9%)	79 (94.0%)	
Surgical	17 (4.1%)	5 (6.0%)	
PICC dwell time (days)	18 (2,74)	17 (1,63)	0.148
Side of PICC insertion, n (%)			0.577
Right	385 (94.8%)	78 (96.3%)	
Left	21 (5.2%)	3 (3.7%)	
PICC insertion site, n (%)			0.234
Upper limbs	336 (81.4%)	73(88.0%)	
Head and neck	7 (1.7%)	2 (2.4%)	
PICC material, n (%)			0.883
Silastic	287 (69.5%)	58 (69%)	
Polyurethane	126 (30.5%)	26 (30%)	
Nonselective removal	31 (7.5%)	15 (18.1%)	0.002

**Table 2:** Comparison of infant PICC insertion site between central and noncentral tip position.

PICC insertion site, n (%)	Central (n=413)	Non-central (n=83)
Upper limbs	336	73
Waist	214(63.6%)	46(63%)
Cubital	34(10.1%)	8(11%)
Cephalic vein	45(13.4%)	9(12.3%)
Axillary vein	43(12.8%)	10(13.7%)
Lower limbs	70	8
Saphenous	61(87.1%)	7(87.5%)
Femoral vein	9(12.9%)	1(12.5%)
Head and neck	7	2
Jugular vein	5 (71.4%)	1(50%)
Posterior auricular vein	2 (28.6%)	1(50%)

**Table 3:** Reason for nonselective removal of PICCs.

Complications	Numbers, n (%)
Occlusion	15 (3.0%)
Displacement	5 (1.0%)
Infection	7 (1.4%)
Phlebitis	3 (0.6%)
Leakage	10 (2.0%)
Pleural effusion	3 (0.6%)
Breaks	3 (0.6%)

**Table 4:** Univariable logistic analysis for PICCs complications.

	OR	95%CI	p
Gestational age	0.860	(0.744,0.994)	0.041
Birth weight <1500 g	1.291	(0.690,2.417)	0.425
Boy	0.721	(0.393,1.324)	0.292
Surgery	1.586	(0.451,5.577)	0.472
Left side	2.063	(0.673,6.326)	0.205
Non-upper limbs insertion site	0.485	(0.169,1.394)	0.179
Silastic	1.447	(0.714,2.932)	0.305
PICC dwell time	0.980	(0.952,1.009)	0.174
Non-central	2.718	(1.393,5.303)	0.003

**Table 5:** Multivariable logistic analysis for PICCs complications.

	OR	95%CI	p
Gestational age	0.830	(0.687,1.003)	0.053
Birth weight <1500 g	1.030	(0.436,2.437)	0.946
Boy	0.582	(0.294,1.154)	0.121
PICC dwell time	0.979	(0.948,1.010)	0.184
Non-central tip position	2.621	(1.258,5.461)	0.010
